# Addictive behaviors in schizophrenic patients: Descriptive and analytical study

**DOI:** 10.1192/j.eurpsy.2023.2320

**Published:** 2023-07-19

**Authors:** W. Bouali, H. Babba, F. Zaouali, M. Kacem, S. Brahim, L. Zarrouk

**Affiliations:** Psychiatrie, Faculty of Medicine of Monastir, Mahdia, Tunisia

## Abstract

**Introduction:**

The association of an addictive disorder (harmful use, abuse or dependence) with a schizophrenic disorder is the rule. Genetic vulnerability and social and economic factors are common to both disorders.

**Objectives:**

determine the impact of addictive behavior on patients suffering from schizophrenia.

**Methods:**

A descriptive and analytical retrospective study of 150 patients with schizophrenia hospitalized in the psychiatry department of Taher Sfar University Hospital in Mahdia from January 2017 to December 2021.

**Results:**

The average age of the patients was 39.8 ± 11.23 years with a predominance of the age group 36-45 years (38.4%). All of the patients were male. Three quarters of the patients (75.5%) were consumers of psychoactive substances (PSA): nearly three quarters (72.8%) were dependent on tobacco, more than a third (39.7%) were dependent on alcohol, more than a quarter (29.1%) dependent on cannabis and almost a quarter (26.5%) dependent on other SPAs. Criminal history, suicide attempts and hospitalization in psychiatry were significantly more frequent in patients who consumed SPA than those who did not consume (39.5% vs 8.1%; p=0.008, 17.5% vs 2.7%; p=0.02, 89.5 % vs 75.7%; p=0.03, respectively). Patients who consumed SPA had significantly more positive signs of schizophrenia (51.8% vs 10.8%; p=0.001) and were significantly less observant to treatment (55.3% vs 16.3%; p=0.001) than those who did not consume. Hetero-aggressiveness, self-aggressiveness and job change were significantly more frequent in patients with addictive behaviors than those without addiction (86.8% vs 48.7%; p=0.001, 23.7% vs 2.7%; p= 0.004, 14.9% vs 0%; p=0.015, respectively). Multivariate analysis revealed that criminal history, hetero-aggressiveness, predominant positive symptomatology and work stoppage were the factors independently associated with SPA consumption in patients with schizophrenia in our study (β=14.7 95% CI 3.23–67.01, p=0.001, β=0.099, 95% CI 0.03–0.31, p=0.001, β=7.18, 95% CI 2.09–24.67, p=0.002, β=5.24 95% CI 1.27–21.7; p=0.02, respectively).

**Conclusions:**

According to our study, addictive comorbidities concern three quarters of our patients. They expose them to a higher risk of legal problems, hetero-aggressiveness, predominance of positive signs and instability at work. These results encourage the development of methods for early diagnostic identification of addictive behavior comorbid with schizophrenia as well as integrated care combining psychiatric and addictological care.

**Disclosure of Interest:**

None Declared

